# Management delays in patients with squamous cell cancer of neck node(s) and unknown primary site: a retrospective cohort study

**DOI:** 10.1186/s40463-017-0217-z

**Published:** 2017-05-08

**Authors:** Kevin Martell, Joanna Mackenzie, Warren Kerney, Harold Yeehau Lau

**Affiliations:** 1 Division of Radiation Oncology, Tom Baker Cancer Centre, Calgary, AB Canada; 2 Department of Oncology, University of Calgary, 1331 29 Street Northwest, Calgary, AB T2N 4 N2 Canada; 3 Edinburgh Cancer Centre, Western General Hospital, Edinburgh, Scotland UK; 4 Calgary Zone, Alberta Health Services, Calgary, AB Canada

**Keywords:** Unknown primary, Neck node, Squamous cell carcinoma, Head and neck, Diagnostic workup, Treatment delay, Diagnostics

## Abstract

**Background:**

We aim to characterize the workup received by and identify any delays to diagnosis or treatment in patients referred to a tertiary cancer centre with the diagnosis of squamous cell carcinoma in neck node(s) and no identifiable primary (SCCNIP).

**Methods:**

Over 1 year, 68 patients were initially referred to the Head and Neck clinic with a label of “primary unknown”. After extensive workup, 29 of the 68 patients were found to have pathologically confirmed SCCNIP. For these 29 patients, imaging tests, biopsies, examinations and times to treatment were reviewed and compared to 145 patients referred for known primaries.

**Results:**

In 21/29 (72%) patients, ultrasound was ordered prior to biopsy or referral. After referral, the first imaging test used was CT neck in 28 patients and PET/CT in 1 patient.

Median time from referral to primary identification (*n* = 23) or workup completion (*n* = 6) were 16 (range: 0-48) and 36 (17-82) days respectively. Median time from referral to treatment was 55 (27-90; *n* = 26) days and was longer than those referred for known primaries (48 days; 20-162; *p* < 0.001). Across all patients, median time between first diagnostic imaging test and pathologic diagnosis were 20.5 and -8.0 days (*p* < 0.0001) in patients receiving ultrasound and CT, respectively.

**Conclusions:**

In our cohort, delays to management were linked to community use of ultrasound and scheduling of both CT and PET/CT after thorough head and neck examination in patients with SCCNIP.

## Background

Head and neck malignancies of unknown primary are unique malignancies in their workup and treatment [1–6]. These patients often receive unnecessary tests which can delay diagnosis and treatments. There is also additional emotional distress in patients who receive delays in treatment for sequential tests which fail to give additional diagnostic information.

For true unknown primaries, Grau et al. [7] have demonstrated that the addition of bilateral neck irradiation in treatment of these malignancies doubles 5 year control rates. Conversely, in head and neck malignancies with known primaries, bilateral neck irradiation often increases toxicity without increasing disease control. Hence, a diagnosis of “unknown primary” should only be made after an extensive workup [1, 6, 8].

Initial literature reviews showed no other studies analyzing the delays in treatment resulting from workup performed for head and neck malignancies after referral to a tertiary cancer centre. However, several strategies for workup of unknown primaries have been suggested [2, 3, 5, 9]. Unfortunately, these often included restricted tests or specialized invasive procedures such as tonsillectomy which Randall et al. [10] showed can provide diagnosis for up to 20% of these patients. Also, more recently, both Reglink et al. [11] and Rudmik et al. [12] have shown the utility of FDG-PET (Fluorodeoxyglucose positron emission tomography) imaging in these malignancies.

Hence we seek to characterize the workup received by patients with metastatic squamous cell carcinoma (SCC) to neck nodes and unknown primary at our centre, quantify the delay from referral to treatment as compared to known primary patients and identify any potential delays to diagnosis or treatment caused by use of unnecessary tests either before or after referral.

## Methods

### Setting

The Tom Baker Cancer Centre (TBCC) is the tertiary cancer centre for southern Alberta, Canada and has a catchment population of approximately two million. Head and neck cancer patient referrals to this centre primarily come from family practitioners. Otolaryngologists, and oromaxillofacial surgeons. The main requirement for referral is a pathologic diagnosis of malignancy. After referral, a multidisciplinary team involving otolaryngologists, radiation oncologists and medical oncologists will review the patient in clinic, perform a complete head and neck exam including nasopharyngoscopy and arrange for any required additional investigations and decide on management recommendations.

### Study population

**Fig. 1:**
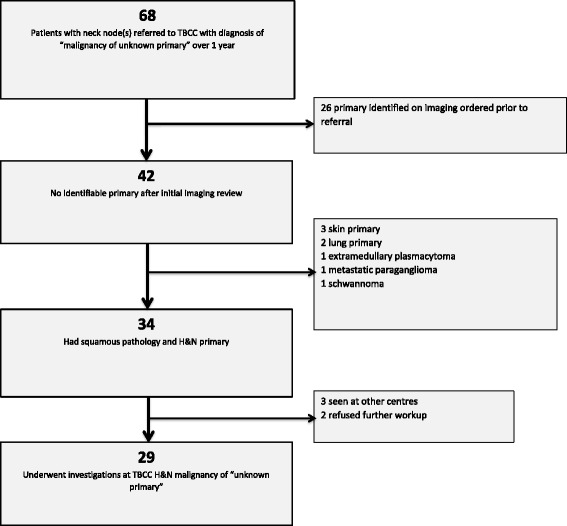
Flow diagram of patients referred to TBCC for head and neck malignancy of unknown primary

Between January 1 and December 31, 2014, a total of 286 patients were referred to the TBCC multidisciplinary head and neck clinic for consideration of head and neck malignancies. For purposes of this study the 68 patients referred with a diagnosis of “head and neck malignancy of unknown primary” were then retrospectively reviewed by two independent physicians. Patients were then excluded from analysis if they were treated elsewhere (3), refused further workup (2), did not have squamous pathology (3), had a non-head and neck primary (5; 3 skin and 2 lung) or a primary lesion was identified through imaging or clinical examination performed prior to referral (26). This left 29 patients with pathologically confirmed squamous cell carcinoma diagnosed on cervical lymph node fine needle aspirate (25) or core/excisional biopsy (4) which forms the cohort of our analysis [Fig. 1]. To quantify the delay to treatment resulting from workup of unknown primaries, an additional, subsequent population consisting of the 145 patients referred to TBCC during the same time period and having known head and neck SCC with identifiable primaries at the time of referral was used to perform a comparative analysis.

### Ethical considerations and data collection

This study is a retrospective cohort analysis. It was designed for purposes of quality review and patient outcome enhancement. Ethical review was performed by an independent third party reviewer using the institutionally approved method described by Hagen et al. [13]. A prospectively collected database containing all referrals to the Tom Baker Cancer Centre multidisciplinary head and neck cancer treatment team was then used to identify patients with initial diagnosis of unknown primary. Retrospective chart reviews of both the local electronic medical record and the provincial electronic health record which houses results for all diagnostic tests performed in Alberta was then undertaken for these patients (including those ordered by a primary care provider). All subsequent diagnostic investigations, appointment and treatment dates were then collected for each patient. These tests were then assessed and a test was considered to have given a diagnosis when two independent physician reviewers agreed that sufficient evidence to identify the primary malignancy was acquired.

### Statistical methods

The primary study cohort was characterized using descriptive statistics. Times from referral to TBCC to diagnosis, then confirmation of diagnosis for each patient were then calculated. Additionally, days to appointment and treatment were calculated. For calculations of delays caused by inappropriate workup, the time from the inappropriate test (eg ultrasound) to pathologic confirmation of disease were calculated. For comparison, time between pathologic diagnosis and the first imaging test was calculated for patients with only appropriate diagnostic workups was calculated. The Shapiro-Wilks test was employed on all calculated time differences for determination of normality. For comparative analysis with the reference group, the Mann-Whitney test was employed to determine significance between medians for times to treatment. Two tailed *p*-values of <0.05 were then accepted as representing statistical significance. All data was analyzed using the R-programming language version 3.1.1 (www.r-project.org).

## Results

### Unknown primary cohort

**Fig 2:**
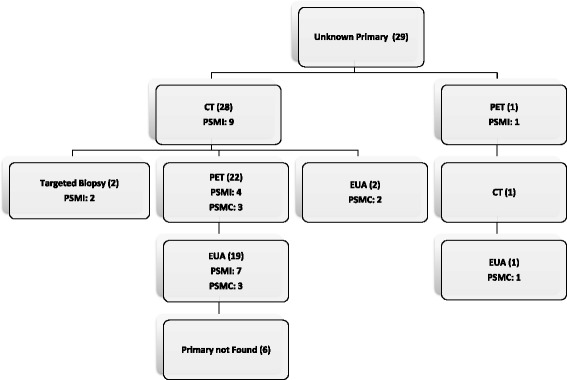
Flow chart of patient investigations for head and neck malignancies of unknown primaries. Indicated are the numbers of patients receiving each investigation in brackets. PSMI: Primary site of malignancy identified. This is the number of patients for which the primary was identified by the corresponding diagnostic test. PSMC: Primary site of malignancy confirmed. This is the number of patients for which the current test confirmed a previously identified lesion

A total of 29 patients had confirmed unknown primary site of malignancy at the time of diagnosis. All 29 underwent CT scan, 23 received PET scans and 19 required EUAs. As shown in Fig. 2, the first investigation following referral was CT neck in 28 patients and PET/CT in 1 patient. From these investigations, 10 (34%) patients had a primary site of malignancy identified (9 from CT and 1 from PET). Of the remaining 18 patients 2 patients underwent targeted biopsies and were subsequently diagnosed with a salivary gland and a tonsillar primary pathologically; 4 patients were diagnosed on subsequent PET scan; and 7 were diagnosed after examination under anesthetic (EUA). This left six patients (21%) where CT, PET and EUA showed no evidence of a primary site. These patients were given a final diagnosis of ‘head and neck carcinoma of unknown primary’ and represent 2% of referrals to our multidisciplinary head and neck clinic.

Of the 29 patients, the referring physicians were otolaryngologists (14), general practitioners (14) and 1 was referred by an oncologic surgeon.

Review of the initial community based workup of these patients revealed that 21 patients (72%) had undergone ultrasound (USS) for consideration of neck mass before being diagnosed with a malignancy. For these, median time from USS to FNA (fine needle aspirate) or core biopsy was 20 days (range: 0-51). Initial pathology was via fine needle aspirate and core biopsy in 25 and four patients respectively.

p16 is a cyclin dependent kinase inhibitor and a surrogate marker for HPV related malignancies [14]. Fourteen patients had p16 status reported initially (4/4 core biopsies, 8/25 FNA). Of these 12 were p16 positive and two were p16 -ve. A further 11 went on to have repeat pathology or review and were p16 positive. In four patients p16 testing was not performed because their original biopsy specimens were inadequate and no further positive biopsies were made.

Median time from referral to TBCC to completion of workup and diagnosis of “Head and neck SCC of unknown primary” was 36 days (range: 17-82; *n* = 6). Median time from referral to identification of a primary was 16 days (0-48; *n* = 23). Of the 23 primary sites of malignancies identified, 12 (52.2%), 8 (34.8%) and 2 (8.7%) of these cancers were tonsillar, base of tongue and nasopharyngeal primaries. There was one (4.3%) salivary gland tumor. 9 (39%), 9 (39%), 3 (13%) and 2 (8%) were T1, T2, T4 and TX malignancies. 26 (90%) patients received treatment (1 patient declined and 2 had yet to start before the data were locked). Median time from diagnosis to treatment was 36 (14-84) days. Twenty-five patients (86%) went on to have chemoradiotherapy. Other treatments included 1 (3%) patient having surgery, 1 (3%) with surgery followed by radiotherapy and 1 (3%) with radiotherapy alone.

### Comparison

**Fig. 3:**
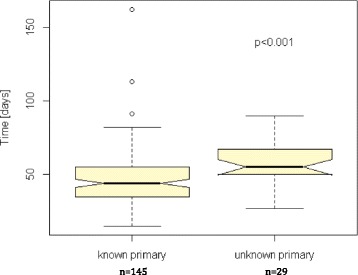
Notched box plot of time (days) between the patient referral being received by the Tom Baker Cancer Centre and patient starting treatment with radiotherapy or surgery for all patients with known and unknown primary site of malignancy at the time of referral

**Fig 4:**
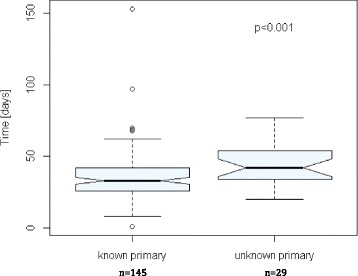
Notched box plot of time from initial appointment at the Tom Baker Cancer Centre to the start of treatment with radiotherapy or surgery for all patients with known and unknown primary site of malignancy at the time of referral

Median number of days between referral to TBCC and treatment was 48 (20-162) vs 55 (27-90); *p* < 0.001 [Fig. 3] and from appointment to treatment was 34 (12-153) vs 42 (20-77); *p* < 0.001 [Fig. 4] for patients with known (145) and unknown primaries (29) at the time of referral respectively.

The largest delays from referral to treatment were noted in the known primary cohort. In explanation of this, 3 patients initially refused treatment resulting in times from referral to treatment of 162, 113 and 76 days. An additional 7 patients in this cohort required additional workup resulting in times from referral to treatment between 67 and 91 days. Use of ultrasound as a first diagnostic imaging investigation again led to a median of a 20.5 [(-53) to (+359); *n* = 62] day delay to pathologic diagnosis. With patients undergoing CT/MR, pathologic diagnosis was made a median of 8 days before their imaging investigation [-8.0; (-77) to (+202); *n* = 80; *p* < 0.0001].

## Discussion

We identified several sources of delay in patients referred to a Canadian tertiary cancer centre for head and neck malignancies with unknown primary. Median time to pathologic diagnosis of malignancy was significantly greater (20.5 days vs -8.0) in patients undergoing community requested ultrasound for confirmation of a neck mass when compared to those having only appropriate workup ordered. Additionally, patients referred for unknown primaries undergo many diagnostic tests including PET, CT and EUA after referral which results in a statistically significant increase in time from referral to treatment when compared to patients with identifiable primaries (48 vs 55 days).

All 29 patients went on to have CT scans and 23 had PET scans. In the 3 patients who underwent PET after a diagnosis was made on CT, the PET scan confirmed the diagnosis. There were only 6 patients diagnosed via CT who did not go on to have a PET. Of note, our PET detection rate (35% in those receiving PET) is quite similar to those cited elsewhere [12, 15–17]. Our results are also similar to Regelink et al. [11], who analyzed outcomes in 50 patients who received PET, EUA and CT. They identified 16, 12 and 11 primaries respectively. This implies that, in centres where it is readily available and easily accessible, PET scan should be the first test of choice after thorough head and neck examination for these patients. This could lead to reduction in delays to treatment and an additional cost savings from the unnecessary diagnostic CT scans [11, 18].

Furthermore, 22 patients had EUA examination after referral and 1 out of 2 targeted biopsies was of a site that would normally be sampled during EUA. An additional attempt to reduce time to treatment could be made by scheduling PET scan and EUA at the time of triaging. This is supported by Waltonen et al. [14], however careful review of the patient chart would be necessary as many patients triaged as malignancy of unknown primary did have a diagnosis. Finally, it has been shown that transoral robotic surgery in diagnostic workup of unknown primaries can improve rates of primary site detection to 80-90% [19, 20]. As this procedure becomes more popular it may be beneficial to schedule this early in the diagnostic workup and perhaps at the time of EUA [21].

Delays to *pathologic* diagnosis in this cohort were attributable to ultrasound being utilized as a first investigation after presentation to their primary care provider for a neck mass (median time to pathologic diagnosis of 20.5 days vs -8 days when compared to patients having only appropriate workup). On review of current practice guidelines, ultrasound was not identified as a recommended diagnostic procedure [22–26]. It is important to acknowledge that the number of community ultrasounds interpreted as benign lymphadenopathy is not addressed in this analysis and remains unknown. However, over the last decade there has been a substantial increase in HPV related oropharyngeal cancers which often present as painless neck lymphadenopathy [27]. Furthermore, the clinical presentation of a persistent, painless neck node should arouse suspicion for malignancy [28]. Hence, ordering an ultrasound guided FNA as the initial diagnostic procedure may be preferred as it could reduce time to diagnosis [29]. Additional measures could include the creation of a specialized clinic for expedited workup and diagnosis of a neck mass.

When interpreting the results of this study it is important to acknowledge that this is a retrospective cohort analysis and inherent bias from unknown confounders is possible. As a single centre study it may not be generalizable to other jurisdictions. For example, PET/CT is not readily available in all centres. Additionally, there were fewer patients than anticipated with head and neck squamous cell carcinomas of unknown primary at the time of referral. Hence, these numbers should be interpreted with caution until larger, multicentre studies are conducted. Finally, this study does not address whether the treatment delays seen in this cohort affected survival outcomes.

## Conclusions

We present an analysis of the typical workup and delay in time to treatment experienced by patients with squamous cell cancer in a neck node without an identifiable primary. One major source of delay was community delays to diagnosis caused by use of ultrasound. This can be avoided in the future though enhanced primary care education, updating primary care guidelines to specifically address ultrasound as an inappropriate test and suggest ultrasound guided biopsy or introduction of expedited care pathways or creation of dedicated clinics to assist primary care practitioners with assessment. A second source of delay was the waitlist for CT scans. As a large majority of these patients ultimately received PET scans, perhaps if patients referred with biopsy proven malignancy in neck nodes could proceed to expedited PET/CTs, this could shorten delays to diagnosis and treatment but would require additional resources for cancer centres.

## Abbreviations

CT: Computed tomography
EUA: Examination under anaesthesia
FNA: Fine needle aspirate
HPV: Human papilloma virus
MRI: Magnetic resonance imaging
PET: Positron emission tomography
SCC: Squamous cell carcinoma
TBCC: Tom Baker Cancer Centre
USS: Ultrasound

## References

[1] Eisbruch A, Foote RL, O’Sullivan B, Beitler JJ, Vikram B (2002). Intensity-modulated radiation therapy for head and neck cancer: emphasis on the selection and delineation of the targets. Semin Radiat Oncol.

[2] Cianchetti M, Mancuso A, Amdur RJ, Werning JW, Kirwan J, Morris CG (2009). Diagnostic evaluation of squamous cell carcinoma metastatic to cervical lymph nodes from an unknown head and neck primary site. Laryngoscope.

[3] Pavlidis N, Briasoulis E, Hainsworth J, Greco F (2003). Diagnostic and therapeutic management of cancer of an unknown primary. Eur J Cancer.

[4] Nasopharyngeal Cancer Treatment Concensus Guidelines. 2013. http://www.albertahealthservices.ca/info/cancerguidelines.aspx. Accessed 28 Sept 2015.

[5] Mendenhall WM, Mancuso AA, Parsons JT, Stringer SP, Cassisi NJ (1998). Diagnostic evaluation of squamous cell carcinoma metastatic to cervical lymph nodes from an unknown head and neck primary site. Head Neck.

[6] Galloway TJ, Ridge J (2015). Management of Squamous cancer metastatic to cervical nodes with an unknown primary site. J Clin Oncol.

[7] Grau C, Johansen LV, Jakobsen J, Geertsen P, Andersen E, Jensen BB (2000). Cervical lymph node metastases from unknown primary tumours. Results from a national survey by the Danish Society for Head and Neck Oncology. Radiother Oncol.

[8] Guntinas-Lichius O, Peter Klussmann J, Dinh S, Dinh M, Schmidt M, Semrau R (2006). Diagnostic work-up and outcome of cervical metastases from an unknown primary. Acta Otolaryngol.

[9] Calabrese L, Jereczek-Fossa B, Jassem J, Rocca A, Bruschini R, Orecchia R (2005). Diagnosis and management of neck metastases from an unknown primary. Acta Otorhinolaryngol Ital.

[10] Randall D, Johnstone P, Foss RD, Martin PJ (2000). Tonsillectomy in diagnosis of the unknown primary tumor of the head and neck. Otolaryngol Head Neck Surg.

[11] Regelink G, Brouwer J, De Bree R, Pruim J, Van Der Laan BF, Vaalburg W (2002). Detection of unknown primary tumours and distant metastases in patients with cervical metastases: value of FDG-PET versus conventional modalities. Eur J Nucl Med.

[12] Rudmik L, Lau HY, Matthews TW, Bosch JD, Kloiber R, Molnar CP (2011). Clinical utility of PET/CT in the evaluation of head and neck squamous cell carcinoma with an unknown primary: a prospective clinical trial. Head Neck.

[13] Hagen B, O’Beirne M, Desai S, Stingl M, Pachnowski CA, Hayward S (2007). Innovations in the ethical review of health-related quality improvement and research: The Alberta Research Ethics Community Consensus Initiative (ARECCI). Healthc Policy.

[14] Hoffman M, Ihloff A, Gorogh T, Weise J, Fazel A, Krams M (2010). p16(INK4a) overexpression predicts translational active human papillomavirus infection in tonsillar cancer. Int J Cancer..

[15] Fischbein NJ, Caputo GR, Kaplan MJ, Price DC, Singer MI, Dillon WP (1998). Metastatic head and neck cancer: role and usefulness of FDG PET in locating occult primary tumors. Radiology.

[16] Lassen U, Daugaard G, Eigtved A, Damgaard K, Friberg L (1999). 18 F-FDG whole body positron emission tomography (PET) in patients with unknown primary tumours (UPT). Eur J Cancer.

[17] Bohuslavizki KH, Klutmann S, Kröger S, Sonnemann U, Buchert R, Werner J (2000). FDG PET detection of unknown primary tumors. J Nucl Med.

[18] Metastatic malignant disease of unknown primary origin: Diagnosis and management of metastatic disease of unknown primary origin 2010. https://www.nice.org.uk/guidance/cg104. Accessed 28 Sept 2015. 22259823

[19] Durmus K, Rangarajan SV, Old MO, Agrawal A, Teknos TN, Ozer E (2014). Transoral robotic approach to carcinoma of unknown primary. Head Neck.

[20] Mehta V, Johnson P, Tassler A, Kim S, Ferris RL, Nance M (2013). A new paradigm for the diagnosis and management of unknown primary tumors of the head and neck: a role for transoral robotic surgery. Laryngoscope.

[21] Hatten KM, O’Malley BW, Bur AM, Patel MR, Rassekh CH, Newman JG (2017). Transoral robotic surgery-assisted endoscopy with primary site detection and treatment in occult mucosal primaries. JAMA Otolaryngol Head Neck Surg.

[22] Schwetschenau E, Kelley DJ (2002). The adult neck mass. Am Fam Physician.

[23] Fahimi F, Müller O, Hoffmann TK (2013). Neck mass. Merk Man.

[24] Thandar M, Jonas N (2004). An approach to the neck mass. AJOL.

[25] Gleeson M, Herber A, Richards A (2000). Management of lateral neck masses in adults. BMJ.

[26] Pfister DG, Spencer S, Brizel DM, Burtness B, Busse PM, Caudell JJ (2015). Head and Neck Cancers, Version 1.2015. J Natl Compr Canc Netw.

[27] Shack L, Lau HY, Huang L, Doll C, Hao D (2014). Trends in the incidence of human papillomavirus-related noncervical and cervical cancers in Alberta, Canada: a population-based study. C Open.

[28] Haynes J, Arnold KR, Aguirre-Oskins C, Chandra S (2015). Evaluation of neck masses in adults. Am Fam Physician.

[29] Lo C-P, Chen C-Y, Chin S-C, Lee K-W, Hsueh C-J, Juan C-J (2007). Detection of suspicious malignant cervical lymph nodes of unknown origin: diagnostic accuracy of ultrasound-guided fine-needle aspiration biopsy with nodal size and central necrosis correlate. Can Assoc Radiol J.

